# Determinants of Noncompliance to Clinic Appointments and Medications among Nigerian Children with Epilepsy: Experience in a Tertiary Health Facility in Enugu, Nigeria

**DOI:** 10.1155/2016/6580416

**Published:** 2016-02-21

**Authors:** Roland Chidi Ibekwe, Appolos Chidi Ndukuba, Ann Ebele Aronu, Christopher Bismarck Eke, MaryAnn Ugochi Ibekwe, Ngozi Chinyelu Ojinnaka

**Affiliations:** ^1^Department of Paediatrics, University of Nigeria, Enugu Campus, Enugu, Nigeria; ^2^Department of Psychological Medicine, University of Nigeria Teaching Hospital, Ituku-Ozalla, Enugu, Nigeria; ^3^Department of Paediatrics, Ebonyi State University, Abakaliki, Nigeria

## Abstract

*Purpose*. To determine the frequency and determinants of noncompliance to clinic appointment and medication among Nigerian children with epilepsy.* Method*. This is a cross-sectional survey of noncompliance to clinic appointments and medication among 113 consecutive children with epilepsy attending the Paediatric Neurology Clinic of University of Nigeria Teaching Hospital, Enugu, southeastern Nigeria.* Results*. Noncompliance to clinic appointment and medication was 23% and 15.3%, respectively. The major reasons given were lack of finance, clashing with school time, and forgetting to take the drugs. Children whose mothers were less educated and unemployed were more likely to miss clinic appointments. Noncompliance to medication was associated with poor seizure control. Children that were on phenobarbitone were more likely to be noncompliant with medication than those on sodium valproate and/or carbamazepine.* Conclusion*. Missed clinic appointment and medication noncompliance are common among Nigerian children with epilepsy and financial constraint is the most common reason.

## 1. Introduction

Missed appointments and poor drug compliance are two major problems that have confounded medical practitioners for ages. Reasons and factors influencing default from clinic appointments had been reviewed extensively among adults and Caucasians attending different specialty clinics [1–3]. Reasons given for this included forgetting the appointments, being busy, unclear appointment details, nonsatisfaction with caregivers approach, and feeling well [2–4].

Among children with epilepsy where antiepileptic medications are the mainstay of treatment, compliance to medication is an issue and monitoring compliance can be problematic. In developed countries drug levels are used to monitor compliance, though this also has its drawbacks, particularly when patients are on multiple drugs with conflicting pharmacokinetics, as can happen in epilepsy [3, 5]. This is not available in most developing countries including Nigeria.

In spite of the fact that epilepsy constitutes more than 60% of the patients seen in the Paediatric Neurology Clinic of most teaching hospitals in Nigeria [6, 7] and the high default rate reported in most centres [6–8], there is no study to the knowledge of the authors to assess this. This study was therefore undertaken to determine the compliance to clinic attendance and medication among children with epilepsy attending the Paediatric Neurology Clinic of UNTH, Enugu, southeastern Nigeria, and the reasons and associated risk factors.

## 2. Materials and Methods

This was a cross-sectional study conducted at the Paediatric Neurology Clinic of University of Nigeria Teaching Hospital (UNTH), Enugu, southeastern Nigeria. The study population were consecutive children seen between July 2014 and December 2014 at the clinic that had been reviewed by a consultant paediatric neurologist with a primary diagnosis of epilepsy.

Inclusion criteria were children that have been attending the clinic for at least twelve consecutive months and are followed up at one to three monthly intervals. Antiepileptic medications are prescribed in tablet form because of cost and stability and are supplied by the hospital pharmacy. Caregivers/patients are counseled to present used/unused sachets and antiepileptic purchase receipt(s) at follow-up visits.

Those that met the inclusion criteria were assessed for compliance to clinic attendance in the preceding six months by checking their case records and assessing the regularity of clinic attendance. Case definition for default is met if they had missed any clinic appointment(s). Reasons for default were sought for from the patients and or their caregivers. Subjects and/or caregivers were also assessed for compliance to medications since the last clinic visit using count of used/unused drugs and pharmacy record of drugs supplied. Those that forgot their medication containers were not recruited in that visit but were instructed to bring it in subsequent visit when they were recruited. A patient is deemed to be noncompliant if more than five percent of his medication(s) was unused or/and there is record of incomplete purchase or nonpurchase of prescribed drugs. For those who were noncompliant, the reasons were sought and documented.

Information regarding sociodemographic data was obtained from the patients and their caregivers and documented in a proforma; this included sex, age, who the caregivers were, type of family (classified as either monogamy if the father is married to only the mother or polygamy if he is married to more than one wife), place of abode (rural or urban), number of children in the family, the position of the subject in the family, and the occupation and highest educational achievement of both parents based on which the social class of the child was determined using Oyedeji's method [9].

Seizure related factors were obtained from the case records. Information obtained included seizure type and seizure frequency at diagnosis and at the time of the study based on which seizure control was determined. The antiepileptic medication the subjects were receiving and their EEG findings were also documented.

Seizure control was classified into excellent if antiepileptic drugs have been stopped and there has been no seizure; good control means that there has been no seizure in the past one year or more but the child was still receiving antiepileptic medications, while it is partial if the child had one or more seizures during the preceding year but not more than once a month or if the reduction in frequency was between 50% and 80% and poor if the seizure frequency was more than once a month or the reduction in seizure frequency was <50% in line with the criteria used by Iloeje [10].

The Ethics and Research Committee of UNTH approved the study and all subjects were only recruited if they gave their consent after the purpose of the study was explained to them.

Analysis was done using SPSS statistical package version 19.0. Differences in proportions were tested for statistical significance using the chi-square test or Fisher's exact test as appropriate. Significant variables were subjected to logistic regression. Significance level was set at *p* < 0.05, with 95% confidence level.

## 3. Result

### 3.1. Demographic Characteristics

One hundred and thirteen children were recruited consecutively as they attended clinics during the study period.

There were 74 males (65.5%) and 39 females (34.57%), M : F = 1.92 : 1. The mean age of the children was 9.28 ± 4.59 years.

The subjects were evenly distributed across the socioeconomic classes. Majority of the parents were educated: 64 fathers (56.6%) had at least senior secondary education while 66 (58.4%) mothers completed their senior secondary education. The subjects were predominantly of the Ibo tribe, 110 (97.1%), and of monogamous family type, 107 (94.7%). Most of the subjects live with both parents, 85 (75.2%), while 17 (15%) live with only their mothers; others live with grandparents, uncles, aunties, and nonrelatives. Majority of the subjects lived in urban towns 79 (69.9%).

### 3.2. Compliance to Clinic Attendance

Twenty-six (23.0%) children had defaulted from clinic appointment. The most common reasons were financial constraint, 11 (42.31%), and clashing with school period, 8 (30.76%); other reasons were trying alternative treatment, 3 (11.54%), distance from clinic, 3 (11.54%), UNTH workers' strike, 2 (7.69%), and relocation from the city, 1, and in one case parents had assumed the disease had been cured and thus stopped follow-up appointments. Some of the respondents had more than one reason.

### 3.3. Factors Associated with Clinic Default

#### 3.3.1. Patients' Characteristics

Table 1 showed the association between clinic default and some sociodemographic variables.

*Age*. 20% of children younger than 5 years compared to 21.74% of those older than 10 years defaulted. The difference is not significant (*χ* ^2^ = 0.53, *p* = 0.77).

*Gender*. 21.03% of males defaulted as against 15.38% of females (Fisher's exact test, *p* = 0.12).

*Family Size*. 17.41% of those from families with ≤4 children defaulted while for those from families with more than 4 children 30.00% defaulted (*χ* ^2^ = 3.21, *p* = 0.20).

*Birth Order of the Subjects*. 17.3% of first borne children defaulted compared to 35.29% of those whose birth order was ≥5 (*χ* ^2^ = 4.15, *p* = 0.13).

*Family Type*. 23.16% of subjects from monogamous families defaulted compared to 20% of those whose families are polygamous (Fisher's exact test, *p* = 0.68).

*Place of Residence*. 35.29% of rural dwellers defaulted from follow-up compared to 17.72% of urban dwellers (*χ* ^2^ = 4.14, *p* = 0.04).

*Social Class*. 23.08% of those in social class 1 defaulted compared to 46.15% of those in class 5 (*χ* ^2^ = 6.91, *p* = 0.14).

*Mothers' Education*. Among children whose mothers had tertiary education the default rate was 15.38%, compared to children of illiterate mothers whose default rate was 53.85% (*χ* ^2^ = 12.07, *p* = 0.02).

*Fathers' Education*. Default rate among children whose fathers had tertiary education was 16.00% while those whose fathers were illiterate had a default rate of 47.06% (*χ* ^2^ = 7.60, *p* = 0.11).

*Mothers' Occupation*. Two (20%) children whose mothers were professionals or senior public servants defaulted compared to 9 (50%) children whose mothers were unemployed (*χ* ^2^ = 12.11, *p* = 0.02).

*Fathers' Occupation*. While 21.43% of the children whose fathers belonged to occupation class 1 defaulted from follow-up, 50% of those whose fathers were in occupation class 5 defaulted (*χ* ^2^ = 0.69, *p* = 0.07).

#### 3.3.2. Seizure Characteristics

Table 2 shows the association between clinic default rate and some seizure variables.

*Seizure Type*. 20% of children with generalized tonic clonic seizure defaulted compared to 17.65% of those with complex partial seizures. The relationship between seizure type and clinic default was not significant (*χ* ^2^ = 10.62, *p* = 0.22).

*Seizure Control*. Among those with excellent seizure control 18.18% defaulted compared with 37.50% of those with poor seizure control. The relation between seizure control and clinic default was not significant (*χ* ^2^ = 3.75, *p* = 0.29).

*Antiepileptic Medication*. The most commonly prescribed drugs were carbamazepine 41 (36.28%), phenobarbitone 22 (19.45%), and sodium valproate 15 (13.27%). 6.67% of children on sodium valproate and 17.07% of those on carbamazepine defaulted compared to 33.33% of those receiving phenobarbitone (*χ* ^2^ = 16.13, *p* = 0.02).

The other seizure related variables like age at onset of seizures (*χ* ^2^ = 0.62, *p* = 0.92), previous history of status epilepticus (Fisher's exact test, *p* = 0.76), family history of epilepsy (*χ* ^2^ = 2.75, *p* = 0.1), electroencephalographic findings (*p* = 0.47), and presence of other anomalies (*χ* ^2^ = 0.31, *p* = 0.80) were all not associated with compliance to clinic visits.

*Logistic Regression Analysis*. None of the variables had significant independent effect on the rate of default from clinic appointments, as shown in Table 3.

### 3.4. Compliance to Medication

Eighteen children/caregivers (15.9%) were noncompliant with their medication. The reasons given included the following: the drugs were too expensive, 11 (61.1%), they forgot to give/take drugs, 8 (44.4%), and child resisted taking the drugs, 3 (16.7%); other reasons given were that they felt the children had been cured, 2 (11.1%), they stopped on account of perceived side effect of the drug(s), 2 (11.1%), and/or the medication was not available, 1 (5.5%). Many of them had more than one reason.

### 3.5. Factors Associated with Poor Drug Compliance

#### 3.5.1. Patients' Characteristics

Table 4 highlights the association between patients' sociodemographic characteristics and their compliance to medication.

*Age*. 13.33% of children younger than 5 years compared to 16.87% of those older than 5 years were noncompliant (*χ* ^2^ = 0.42, *p* = 0.81).

*Gender*. 13.51% of males were noncompliant as against 20.5% of females (*χ* ^2^ = 0.91, *p* = 0.31).

*Family Size*. 19.05% of those from families with ≤4 children were noncompliant compared to 12% of those from families with more than 4 children (*χ* ^2^ = 1.15, *p* = 0.56).

*Position of Subject among the Children*. While 13.04% of first born children were noncompliant, among those who were ≥5th born 11.76% were noncompliant with their medication (*χ* ^2^ = 1.16, *p* = 0.56).

*Family Type*. 15.74% of the subjects from monogamous families defaulted compared to 20% of those whose families are polygamous (Fisher's exact test, *p* = 0.59).

*Place of Residence*. 8.82% of rural dwellers were noncompliant with their medication compared to 18.99% of urban dwellers (Fisher's exact test, *p* = 0.14).

*Social Class*. 13.51% of children in the upper social class were not compliant while 20.41% of those in lower social classes were noncompliant (*χ* ^2^ = 2.20, *p* = 0.70).

*Mothers' Education*. Among children whose mothers had tertiary education 11.54% were noncompliant, compared to children of illiterate mothers of which 23.08% were noncompliant (*χ* ^2^ = 5.04, *p* = 0.28).

*Fathers' Education*. 16.0% of children whose fathers had tertiary education were noncompliant while among those whose fathers were illiterate noncompliance rate was 23.53% (*χ* ^2^ = 1.28, *p* = 0.87).

*Mothers' Occupation*. 10% of children whose mothers were professionals or senior public servants were not compliant with their medications compared to 11% of children whose mothers were unemployed (*χ* ^2^ = 2.67, *p* = 0.62).

*Fathers' Occupation*. While 14.29% of the children whose father belonged to occupation class 1 were not compliant to their medications, 20% of those whose fathers were in occupation class 5 were not compliant (*χ* ^2^ = 0.70, *p* = 0.95).

#### 3.5.2. Seizure Characteristics

Table 5 highlights the influence of some of the seizure variables and default rate of the subjects on their compliance to medication.

*Seizure Type*. 16.25%, 17.65%, and 12.5% of children with generalized tonic clonic, complex partial, and other seizure types were not compliant with their medication. The relationship between seizure type and compliance to medication was not significant (*χ* ^2^ = 3.54, *p* = 0.90).

*Seizure Control*. Among those with excellent seizure control 9.09% were not compliant with their medication compared with 33.33% of those with poor seizure control that defaulted (*χ* ^2^ = 8.34, *p* = 0.04).

*Antiepileptic Medication*. The rates of noncompliance to the most commonly prescribed antiepileptic drugs were 18.8% (phenobarbitone), 9.76% (carbamazepine), and 0.0% (valproate). The relationship between antiepileptic medication received and rate of noncompliance is statistically significant (*χ* ^2^ = 14.62, *p* = 0.04).

#### 3.5.3. Default Rate

Among those that defaulted from follow-up appointment(s) 33.33% were not compliant with their medications compared to 13.79% of those that did not default (Fisher's exact test, *p* = 0.36).

## 4. Discussion

The missed appointment rate in this study is 23%; a similar study in Saudi Arabia reported a default rate of 47% [3], while rates between 15 and 63% had been reported from South Africa and various studies in the USA [11]. Though the study design may have underestimated those that have been lost to follow-up, it afforded the researchers the opportunity of face-to-face interview with all the parents of the patients. This would not have been possible if medical record had been used. Extensive counseling of parents/patients at every clinic contact by both doctors and nurses in the unit on the need and benefits of regular follow-up visits may have contributed to the relatively low default rate. Though the default rate appears to be low, the target should be to reduce it to insignificant level. Studies had highlighted that customized short message service (SMS) reminder improves clinic attendance and is cost-effective [12].

The most common reasons given for missed clinic appointments were clashing with school time and lack of finance for transport and consultation. This is similar to that reported in Saudi Arabia and the USA [1, 3]. In Nigeria the health insurance has limited coverage, health expenditure is out-of-pocket, and this puts significant strain on family budget. It is thus not surprising that finance is a major impediment to effective health delivery in Nigeria. Ezeala-Adikiabe et al. [13] observed that direct cost of management of adult epilepsy patients in UNTH, Enugu, reaches catastrophic health expenditure level. This is not too different from our experience in paediatric epilepsy management. The timing of the Paediatric Neurology Clinic in UNTH is in the mornings and this clashes with the school period. Ibekwe and Ojinnaka [14] had noted increased school absences among children with epilepsy in this centre. They had advocated more flexible clinic hours and appointments for school children should be in out-of-school hours. This unfortunately has been difficult to implement.

Children that presented from rural areas were more likely to miss clinic appointments. The reasons for this may be multifactorial; most parents living in rural areas are less educated and may not be able to read clinic appointment dates. These people are also on the average of lower socioeconomic class than the urban dwellers; thus the resources required to keep these appointments could be daunting.

Default rate was observed to be low among children of educated mothers and those whose mothers earn sizable incomes. Interestingly the fathers' income and education had no influence on clinic appointment. This may be because in most cases mothers are the ones that bring the patients for clinic appointments; if they are economically empowered they are more able to bring their children for appointments. This finding has been previously corroborated. Deyo and Inui [15] noted that older age group, higher education, and socioeconomic status were the only consistent factors that influenced appointment keeping behaviors among adult patients.

The noncompliance rate to medication of 15% in this centre is within the 12–35% rate reported worldwide [3, 5] but much lower than the 60% observed in Cincinnati, USA, using electronic monitors [16, 17]. Due to technical and logistic limitations, the more objective serum drug levels and electronic monitors could not be done. However the combination of drug count and pharmacy record provided a reasonably accurate insight into the magnitude of the problem. The high cost of medication and forgetting to give or take medications were the most common reasons given for not taking their medication(s). In view of the high financial burden of managing these children in the absence of health insurance, it is not surprising that cost was the most common reason for noncompliance.

In Saudi Arabia [3], they noted a link between compliance to clinic appointment and compliance to medication [4]. This was not confirmed in this study; though children who were compliant with appointment were more likely to adhere to their medication this relation did not reach significant level. This may be because patients and their parents are extensively counseled during every clinic visit on the importance of drug compliance [18]. The efficacy, availability, and affordability of the drugs and the social class of parents are considered before AED are prescribed.

In Iran, Asadi-Pooya [19] reported that compliance was better in children receiving phenobarbitone compared to those on either valproate or carbamazepine. He attributed this to the simpler dosing regimen of phenobarbitone which was prescribed once daily. This differs from the finding in this study where there was high noncompliance rate among children on phenobarbitone. In this centre phenobarbitone, carbamazepine, and valproate are mainly prescribed on BD basis. It is only valproate that is sometimes prescribed thrice a day; thus dosage regimen did not appear to be a significant consideration in compliance.

In conclusion, noncompliance to clinic visits and medication is significant impediment to optimal care of children with epilepsy in Nigeria. Major reasons for this are lack of finance for transportation, consultation and drugs, clashing with school period, and forgetting to take medications. In order to address this the National Health Insurance scheme should be escalated to increase its coverage so as to reduce out-of-pocket health expenditure, clinics should be more flexible and appointments for clinics should be in out-of-school hours, and medications should be clearly written and explained, including in local languages.

## Limitation of Study

Default was based on retrospective review of medical records of those that attended clinics. This would have underreported those lost to follow-up. However it afforded the researchers the opportunity to ascertain reasons for default. Medication compliance was based on drug count and pharmacy record; the more objective serum drug levels and electronic monitors were not done because of technical and logistic constraints.

## Figures and Tables

**Table 1:** Association of noncompliance to clinic appointments and some sociodemographic variables among children with epilepsy.

	Nondefaulters *N* (%)	Defaulters *N* (%)	*χ* ^2^	*p* value
Age group (years)				
≤5	24 (80.00)	6 (20.00)	0.53	**0.77**
6–10	27 (72.97)	10 (27.02)		
>10	36 (78.26)	10 (21.74)		
Gender				
Male	54 (72.97)	20 (27.03)	*∗*	0.12
Female	33 (84.62)	6 (15.38)		
Family size				
1	4 (100.00)	0 (0.00)	3.21	0.20
2–4	48 (81.36)	11 (18.64)		
≥5	35 (70.00)	15 (30.00)		
Position of subject among siblings				
1st	19 (82.61)	4 (17.39)	4.15	0.13
2nd–4th	46 (82.14)	10 (17.86)		
≥5	22 (64.71)	12 (35.29)		
Family type				
Monogamy	83 (76.85)	25 (23.15)	*∗*	0.68
Polygamy	4 (80.00)	1 (20.00)		
Place of abode				
Rural	22 (64.71)	12 (35.29)	4.14	0.04
Urban	65 (82.28)	14 (17.72)		
Mothers' highest education				
University	22 (84.62)	4 (15.38)	12.07	0.02
Diploma/national cert in education	6 (66.67)	3 (33.33)		
Senior secondary	29 (90.63)	3 (9.37)		
Primary/junior secondary	24 (72.73)	9 (27.27)		
No formal education	6 (46.15)	7 (53.85)		
Fathers' highest education attainment				
University	21 (84.00)	4 (16.00)	7.60	0.11
Diploma/national cert in education	10 (90.91)	1 (9.09)		
Senior secondary	24 (80.00)	6 (20.00)		
Primary/junior secondary	23 (76.67)	7 (23.33)		
No formal education	9 (52.94)	8 (47.06)		
Mothers' occupation				
Professionals/senior managers	8 (80.00)	2 (20.00)	12.15	0.02
Intermediate public servants/school teachers and equivalent	22 (95.65)	1 (4.35)		
Junior teachers/drivers/artisans	21 (77.78)	7 (22.22)		
Messengers/labourers/petty traders	27 (79.41)	7 (20.59)		
Unemployed/housewives/students	9 (50.00)	9 (50.00)		
Fathers' occupation				
Professionals/senior managers	22 (78.57)	6 (21.43)	8.69	0.07
Intermediate public servants/school teachers and equivalent	18 (85.71)	3 (14.29)		
Junior teachers/drivers/artisans	22 (68.75)	10 (31.25)		
Messengers/labourers/petty traders	20 (90.91)	2 (9.09)		
Unemployed/students	5 (50.00)	5 (50.00)		
Social class				
1	10 (76.92)	3 (23.08)	6.91	0.14
2	21 (87.50)	3 (12.50)		
3	23 (85.19)	4 (14.81)		
4	26 (72.22)	10 (27.78)		
5	7 (53.85)	6 (46.15)		

^*∗*^Fisher's exact test.

**Table 2:** The association between default from clinic appointments and seizure related variables.

	Nondefaulter *N* (%)	Defaulters *N* (%)	*χ* ^2^	*p* value
Seizure type				
Generalized tonic clonic	64 (80.00)	16 (20.00)	10.62	0.22
Complex partial	14 (82.35)	3 (17.65)		
Simple partial	4 (66.67)	2 (33.33)		
Absence	2 (66.67)	1 (33.33)		
Other seizures	3 (42.86)	4 (57.14)		
Age at onset of epilepsy (years)				
<2	8 (80.00)	2 (20.00)	0.06	0.92
2–5	36 (76.60)	11 (23.40)		
>5	43 (76.79)	13 (23.21)		
Antiepileptic medication				
Carbamazepine	34 (82.93)	7 (17.07)	16.13	0.02
Phenobarbitone	16 (66.67)	6 (33.33)		
Sodium valproate	14 (93.33)	1 (6.67)		
Phenytoin	3 (50.00)	3 (50.00)		
Others	4 (66.67)	2 (33.33)		
Seizure control				
Excellent	9 (81.82)	2 (18.18)	3.75	0.29
Good	20 (83.33)	4 (16.67)		
Partial	43 (79.63)	11 (20.37)		
Poor	15 (62.50)	9 (37.50)		
Previous history of status epilepticus				
No	75 (77.32)	22 (22.68)	*∗*	0.76
Yes	12 (75.00)	4 (25.00)		
Family history of epilepsy				
No	73 (80.22)	18 (19.78)	2.75	0.1
Yes	14 (63.64)	8 (36.36)		
Electroencephalogram finding				
Normal	7 (63.64)	4 (36.36)	*∗*	0.47
Abnormal	30 (75.00)	10 (25.00)		
Associated abnormality				
Absent	73 (79.35)	22 (20.65)	0.31	0.80
Present	14 (77.78)	4 (22.22)		

**Table 3:** Logistic regression of variables that were significantly associated with default from clinics.

	*B*	SE	Wald	Df	Sig	Exp(*B*)
Place of abode	0.622	0.568	1.197	1	0.274	1.862
Maternal education	0.143	0.280	0.260	1	0.610	1.153
Maternal occupation	−0.505	0.314	2.582	1	0.108	0.604
Medication	0.061	0.67	0.829	1	0.363	1.063
Constant	1.335	1.406	0.901	1	0.342	3.801

**Table 4:** The association between noncompliance to medication and some sociodemographic variables.

	Compliant *N* (%)	Not compliant *N* (%)	*χ* ^2^	*p* value
Age group (years)				
≤5	26 (86.67)	4 (13.33)	0.42	**0.81**
6–10	30 (81.08)	7 (18.92)		
>10	39 (84.78)	7 (15.22)		
Gender				
Male	64 (86.49)	10 (13.51)	0.91	0.33
Female	31 (70.49)	8 (20.51)		
Family size				
1	3 (75.00)	1 (25.00)	1.15	0.56
2–4	48 (81.36)	11 (18.64)		
≥5	44 (88.00)	6 (12.00)		
Position of subject among siblings				
1st	20 (86.96)	3 (13.04)	1.16	0.56
2nd–4th	45 (76.27)	11 (23.73)		
≥5	30 (88.24)	4 (11.76)		
Family type				
Monogamy	91 (84.26)	17 (15.74)	*∗*	0.59
Polygamy	4 (80.00)	1 (20.00)		
Place of abode				
Rural	31 (91.18)	3 (8.82)	*∗*	0.14
Urban	64 (81.01)	15 (18.99)		
Mothers' highest education				
University	23 (88.46)	3 (11.54)	5.08	0.28
Diploma/national cert in education	7 (77.78)	2 (22.22)		
Senior secondary	30 (93.75)	2 (6.25)		
Primary/junior secondary	25 (75.76)	8 (24.24)		
No formal education	10 (76.92)	3 (23.08)		
Fathers' highest education attainment				
University	21 (84)	4 (16.00)	1.28	0.87
Diploma/national cert in education	10 (90.91)	1 (9.09)		
Senior secondary	26 (86.67)	4 (13.33)		
Primary/junior secondary	25 (83.33)	5 (16.67)		
No formal education	13 (76.47)	4 (23.53)		
Mothers' occupation				
Professionals/senior managers	9 (90)	1 (10)	2.65	0.62
Intermediate public servants/school teachers and equivalent	19 (82.61)	4 (17.39)		
Junior teachers/drivers/artisans	25 (89.29)	3 (10.71)		
Messengers/labourers/petty traders	26 (76.47)	8 (23.53)		
Unemployed/housewives/students	16 (88.89)	2 (11.11)		
Fathers' occupation				
Professionals/senior managers	24 (85.71)	4 (14.29)	0.70	0.95
Intermediate public servants/school teachers and equivalent	17 (80.95)	4 (19.05)		
Junior teachers/drivers/artisans	28 (87.50)	4 (12.50)		
Messengers/labourers/petty traders	18 (81.82)	4 (18.18)		
Unemployed/students	8 (80)	2 (20)		
Social class				
1	12 (92.31)	1 (7.69)	2.20	0.70
2	20 (83.33)	4 (16.67)		
3	24 (88.89)	3 (11.11)		
4	28 (77.78)	8 (22.22)		
5	11 (84.62)	2 (15.38)		

^*∗*^Fisher's exact test.

**Table 5:** The association between noncompliance to medication and seizure related variables.

	Compliant *N* (%)	Not compliant *N* (%)	*χ* ^2^	*p* value
Seizure type				
Generalized tonic clonic	67 (83.75)	13 (16.25)	3.54	0.90
Complex partial	14 (82.35)	3 (17.65)		
Simple partial	5 (83.33)	1 (16.67)		
Absence	3 (100)	0		
Other seizures	6 (85.71)	1 (14.29)		
Age at onset of epilepsy (years)				
<2	9 (90)	1 (10)	2.51	0.29
2–5	42 (89.36)	5 (10.64)		
>5	44 (78.57)	12 (21.43)		
Antiepileptic medication				
Carbamazepine	37 (90.24)	4 (9.76)	14.62	0.04
Phenobarbitone	18 (81.82)	4 (18.18)		
Sodium valproate	15 (100)	0		
Phenytoin	4 (66.67)	2 (33.33)		
Others	5 (83.33)	1 (16.67)		
Seizure control				
Excellent	10 (90.91)	1 (9.09)	8.34	0.04
Good	23 (95.83)	1 (4.17)		
Partial	48 (85.71)	8 (14.29)		
Poor	16 (66.67)	8 (33.33)		
Previous history of status epilepticus				
No	83 (85.57)	14 (14.43)	*∗*	0.28
Yes	12 (75)	4 (25)		
Family history of epilepsy				
No	76 (83.52)	15 (16.48)	*∗*	1.00
Yes	19 (86.36)	3 (13.64)		
Electroencephalographic finding				
Normal	8 (72.73)	3 (27.27)	*∗*	0.35
Abnormal	35 (87.50)	5 (12.50)		
Associated anomalies				
Absent	79 (84.04)	15 (15.96)	0.20	0.91
Present	15 (83.33)	3 (16.67)		
Default clinic appointment				
Yes	12 (66.67)	6 (33.33)	*∗*	0.36
No	75 (86.21)	12 (13.79)		

^*∗*^Fisher's exact test.
